# 4-Acetyl-Antroquinonol B Suppresses SOD2-Enhanced Cancer Stem Cell-Like Phenotypes and Chemoresistance of Colorectal Cancer Cells by Inducing hsa-miR-324 re-Expression

**DOI:** 10.3390/cancers10080269

**Published:** 2018-08-10

**Authors:** Oluwaseun Adebayo Bamodu, Ching-Kuo Yang, Wei-Hong Cheng, David T.W. Tzeng, Kuang-Tai Kuo, Chun-Chih Huang, Li Deng, Michael Hsiao, Wei-Hwa Lee, Chi-Tai Yeh

**Affiliations:** 1 Department of Hematology and Oncology, Cancer Center, Taipei Medical University-Shuang Ho Hospital, New Taipei City 23561, Taiwan; 16625@s.tmu.edu.tw (O.A.B.); 13520@s.tmu.edu.tw (W.-H.C.); 2 Department of Medical Research & Education, Taipei Medical University-Shuang Ho Hospital, New Taipei City 23561, Taiwan; 3 Division of Colorectal Surgery, Department of Surgery, Mackay Memorial Hospital, Taipei City 110, Taiwan; yangchingkao@yahoo.com.tw; 4 School of Life Sciences, The Chinese University of Hong Kong, Hong Kong 153254, China; allqwdd@gmail.com; 5 Division of Thoracic Surgery, Department of Surgery, School of Medicine, College of Medicine, Taipei Medical University, Taipei City 110, Taiwan; doc2738h@gmail.com; 6 Division of Thoracic Surgery, Department of Surgery, Shuang Ho Hospital, Taipei Medical University, New Taipei City 23561, Taiwan; 7 Department of Appiled Chemistry, Chaoyang University of Technology, Taichung 41147, Taiwan; john@newbellus.com.tw; 8 Beijing Bioprocess Key Laboratory, College of Life Science and Technology, Beijing University of Chemical Technology, Beijing 100029, China; dengli@mail.buct.edu.cn; 9 Amoy-BUCT Industrial Bio-Technovation Institute, Amoy 361022, China; 10 Genomics Research Center, Academia Sinica, Taipei 11529, Taiwan; mhsiao@gate.sinica.edu.tw; 11 Department of Pathology, Taipei Medical University-Shuang Ho Hospital, New Taipei City 23561, Taiwan; 12 Department of Pathology, School of Medicine, College of Medicine, Taipei Medical University, Taipei City 110, Taiwan

**Keywords:** 4-AAQB, SOD2, hsa-miR-324, colon cancer stem cells, chemoresistance, chemosensitivity

## Abstract

Background: Colorectal cancer (CRC) remains a leading cause of cancer-related morbidity and mortality in both sexes globally. This is not unconnected with the heterogeneity and plasticity of CRC stem cells (CRC-SCs) which stealthily exploit the niche-related and (epi)genetic factors to facilitate metastasis, chemoresistance, tumor recurrence, and disease progression. Despite the accumulating evidence of the role of dysregulated microRNAs in malignancies, the therapeutic efficacy of pharmacological-targeting of CRC-SC-associated microRNAs is relatively under-explored. Experimental approach: In this present study, we employed relatively new bioinformatics approaches, analyses of microarray data, Western blot, real-time polymerase chain reaction (RT-PCR), and functional assays to show that hsa-miR-324-5p expression is significantly suppressed in CRC cells, and inversely correlates with the aberrant expression of SOD2. Results: This converse hsa-miR-324-5p/SOD2 relationship is associated with enhanced oncogenicity, which is effectively inhibited by 4-acetylantroquinonol B (4-AAQB), as evidenced by inhibited cell viability and proliferation, as well as attenuated migration, invasion, and clonogenicity in 4-AAQB-treated DLD1 and HCT116 cells. Interestingly, 4-AAQB did not affect the viability and proliferation of normal colon cells. We also showed that 4-AAQB-induced re-expression of hsa-miR-324-5p, akin to short-interfering RNA, reduced SOD2 expression, correlates with the concurrent down-regulation of SOD2, N-cadherin, vimentin, c-Myc, and BcL-xL2, with concomitant up-regulation of E-cadherin and BAX2 proteins. Enhanced expression of hsa-miR-324-5p in the CRC cells suppressed their tumorigenicity in vitro and in vivo. Additionally, 4-AAQB synergistically potentiates the FOLFOX (folinate (leucovorin), fluorouracil (5FU), and oxaliplatin) anticancer effect by eliciting the re-expression of SOD2-suppressed hsa-miR-324, and inhibiting SOD2-mediated tumorigenicity. Conclusion: Our findings highlight the pre-clinical anti-CSC efficacy of 4-AAQB, with or without FOLFOX in CRC, and suggest a potential novel therapeutic strategy for CRC patients.

## 1. Introduction

Colorectal cancer (CRC) is the third most commonly diagnosed malignancy and ranks as the fourth leading cause of cancer-related deaths in both sexes, globally [1]. In fact, a global estimate of 1,678,127 colorectal cancer cases, has been predicted for the year 2020 [2]. With a 5-year relative survival rate is ≥90% in stage I disease, and ~10% in stage IV disease, the therapeutic mainstay of CRC therapy is surgery, neoadjuvant radiotherapy in rectal cases, and adjuvant chemotherapy for advanced stage and high-risk stage II colon cancer [3].

Over the last decade, a decline in the incidence and mortality rates of CRC has been recorded in high-income countries; conversely, these rates continue to rise in low-resourced nations. This disparity in rates may be attributed to early screening, improved diagnostic techniques, and better therapeutic strategies in the former, and senilization, increased embrace of a Western lifestyle, late diagnosis, the excessive cost of conventional therapy in the latter [4,5], thus necessitating the discovery and/or development of a relatively cheaper and more accessible therapeutic strategy. Similarly, the cumulative evidence of the presence and implication of a small subset of the CRC bulk-termed CSCs in the tumorigenicity, oncogenicity, tumor progression, and disease recurrence of CRC [6,7], further begs the case for a novel therapeutic option with CSCs—targeting potentials, high curative efficacy, and low drug-associated toxicity.

In an earlier work by our team, we demonstrated that 4-acetylantroquinonol B (4-AAQB), a mycelial isolate of *Antrodia camphorata*, a common Taiwanese camphor tree mushroom with a broad range of documented bioactivities, effectively disrupts essential oncogenic signaling pathways such as the Lgr5/Wnt/β-catenin, JAK-STAT, and non-transmembrane receptor tyrosine kinase signaling pathways, inhibits the acquisition of the CRC-stem cell (SC) phenotype, down-regulates the expression and/or activities of stemness-associated genes including *ALDH1*, attenuates tumor aggression, and accentuates chemosensitivity in CRC cells [8]. Consistent with the growing evidence that microRNAs (miRNAs) are deregulated in several human cancer types including breast cancer, lung cancer, and colon cancer, potentially serving as screening and diagnostic factors [9,10], as well as Leufkens et al.’s prospective cohort-nested case-control study (1064 CRC cases, 1064 matched controls), which provided evidence for the involvement of biomarkers of oxidative stress in the development of CRC [11], as a sequela to our previously published work, in this present study, we continued exploring the therapeutic potential of 4-AAQB in CRC, particularly in the context of the probable regulatory role of 4-AAQB on the epigenetic landscape and modulation of oxidative stress in CRC.

In humans, superoxide dismutases (SODs), with three subtypes, namely SOD1/ Cu,Zn-SOD, SOD2/Mn-SOD, and SOD3/extracellular SOD, constitute a principal component of the antioxidant defense system, and are also known as the antioxidases. SOD2 is a 96 kDa homotetramer nuclear-encoded mitochondrial manganese (Mn)-containing antioxidant enzyme. Through its interaction with, and binding to superoxide byproducts of oxidative phosphorylation, SOD2 is able to convert them into diatomic oxygen and hydrogen peroxide (H_2_O_2_). The Mn at the SOD2 active site catalyzes this disproportionization of superoxide anion to oxygen and H_2_O_2_, akin to SOD1 and SOD3 [12]. Despite documented association between mutations in the SOD2 gene and several pathologies, including sporadic motor neuron disease, idiopathic dilated cardiomyopathy (IDC), premature aging (progeria), and cancer, the role of SOD2 in cancer cells, such as in CRC cells, is not fully understood. While there are divergent views on the actual role of SOD2 in cancer, there is growing evidence that the loss of SOD2 function may not facilitate metastatic disease progression as previously thought; rather, there is an indication from experimental and epidemiological studies that enhanced SOD2 expression levels correlate with metastatic malignantization and disease progression in various cancer types [13]. It has been shown that the up-regulation of SOD2 protein expression is characteristic of certain cancers, including cervical and salivary adenoid cystic carcinoma [14,15]. 

Deregulation of epigenetic factors and patterns, such as aberrant DNA methylation, histone tail modification, and non-coding RNA (ncRNA) regulation, are implicated in loss of cell identity and contribute to several human pathologies, including cancer. Recent advances in the field of epigenetics are coupled with accumulating evidence of the critical role of non-coding RNAs, particularly miRNA expression and/or activity in oncogenicity [16]. miRNAs are approximately 22 nucleotide (nt) long evolutionarily conserved small non-coding RNAs (ncRNAs) with the ability to elicit endogenous post-transcriptional silencing of its target genes based on complementarity with target sites in the 3′-untranslated regions (3′-UTRs) of the target gene/messenger RNA (mRNA) [16]. There is documented correlation or association between down-regulated miRNA expression, and tumor initiation or metastatic disease progression [17,18], however, there is a dearth of information regarding SOD2-regulated miRNA(s) in CRC, and regarding how the epigenetic modulation of SOD2 contributes to the CSC-like and metastatic phenotype of CRC cells. 

Against the background of the conflicting data on SOD2 in malignancies, in this present study, we investigated the role of SOD2 in CRC, as well as, if and how 4-AAQB affects SOD2 expression profile in CRC. Additionally, considering the suggested cancer-related bivalence of SOD2, we also probed for probable epigenetic undertone to SOD2-associated phenotype and likely SOD2-attenuating 4-AAQB therapeutic activity in CRC. Consequently, herein, we provide evidence that hsa-miR-324 interacts with SOD2, and that the aberrant expression of SOD2 enhances the oncogenicity and cancer stem cell-like phenotype of CRC cells and the therapeutic effect of 4-AAQB in these cells is mediated by inducing re-expression of SOD2-suppressed hsa-miR-324.

## 2. Results

### 2.1. A Reciprocal Down-Regulation of SOD2 and hsa-miR-324-5p Gene Expression Exists in Various Human Cancer Types, Including CRC Cells

**Figure 1:**
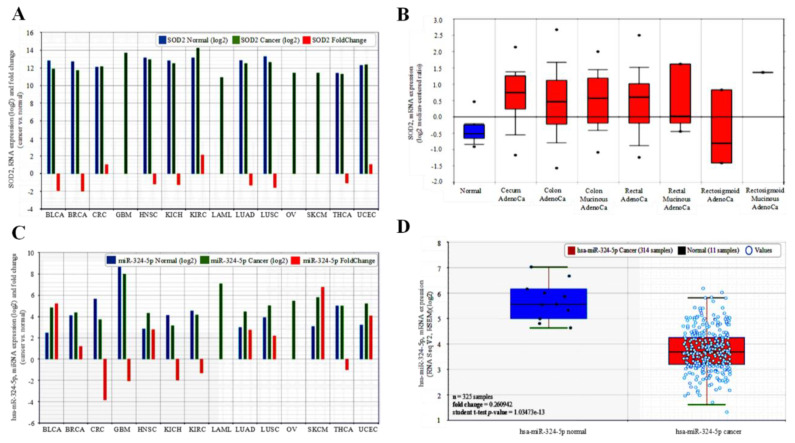
SOD2 is aberrantly expressed in malignancies and correlates with the suppression of hsa-miR-324 expression. (**A**) Comparative analyses of the SOD2 expression levels in normal–cancer tissue pairs of the TCGA 14 cancer types—pancancer cohort dataset using the starBase v2.0 software algorithms. (**B**) SOD2 is up-regulated in colon and rectal adenocarcinoma, CRC samples compared to normal samples (fold change = 1.05, student *t*-test *p*-value = 0.59432). (**C**) Comparative analyses of the hsa-miR-324-5p expression levels in normal–cancer tissue pairs of the TCGA 14 cancer type—pancancer cohort dataset. (**D**) Decreased levels of hsa-miR-324-5p in cancer compared to normal samples (fold change = 0.26, student *t*-test *p*-value = 1.035 × 10^−13^).

To characterize the role of SOD2 in human cancer, we examined the expression profile of SOD2 in matched pairs of tumor and adjacent non-tumor tissues in 14 different human cancer types from the cancer genome atlas (TCGA) PANCANCER dataset with SOD2 data (*n* = 5599). Compared to its expression in adjacent non-tumor tissues, hsa-miR-324-5p expression was significantly down-regulated in bladder urothelial carcinoma (BLCA, ~0.53-fold), breast invasive carcinoma (BRCA, ~0.5-fold), head and neck squamous cell carcinoma (HNSC, ~0.83-fold), kidney chromophobe cell carcinoma (KICH, ~0.77-fold), lung adenocarcinoma (LUAD, ~0.77-fold), lung squamous cell carcinoma (LUSC, ~0.55-fold), and thyroid carcinoma (THCA, ~0.91-fold), but conversely, up-regulated in colorectal cancer (CRC, ~1.0-fold), kidney renal clear cell carcinoma (KIRC, ~2.2-fold), and uterine corpus endometrial carcinoma (UCEC, ~1.1-fold), while no comparative non-tumor data was available for glioblastoma (GBM), acute myeloid leukemia (LAML), ovarian carcinoma(OV), and skin cutaneous melanoma (SKCM) (Figure 1A). The SOD2 expression data for CRC was corroborated by that which was obtained from the analysis of the TCGA CRC dataset (*n* = 237) using the Oncomine platform (https://www.oncomine.org), with a 8.47-fold (*n* = 22, *p* = 5.55 × 10^−10^), 1.93-fold (*n* = 101, *p* = 1.92 × 10^−13^), 1.88-fold (*n* = 60, *p* = 2.88 × 10^−10^), and 1.45-fold (*n* = 6, *p* = 6.25 × 10^−4^) upregulation of SOD2 expression level was observed in the colon mucinous adenocarcinoma, colon adenocarcinoma, rectal adenocarcinoma, and rectal mucinous adenocarcinoma compared to the non-tumor ‘normal’ colorectal tissues (Figure 1B). Furthermore, we employed a bioinformatics approach to systematically screen for miRNAs that interact with SOD2, we sorted them out by interaction propensity, sequence complementarity, and broad conservation across species based on data from TargetScanHuman release 7.1 (http://www.targetscan.org/vert_71/), and miRDB (http://www.mirdb.org/). We observed high interaction propensity, broad conservation, and good complementarity between the 5′ end of hsa-miR-324-5p and the 3′ end of SOD2. In parallel analyses of the TCGA datasets, in comparison with SOD2, we observed a reciprocity in the expression profile of hsa-miR-324-5p for CRC and KIRC only in the miRNA-relevant PANCANCER dataset (*n* = 5613); such that hsa-miR-324-5p was significantly suppressed in CRC (~0.26-fold), KIRC (~0.77-fold), GBM (~0.48-fold), KICH (~0.50-fold), and THCA (~0.91-fold) compared to the non-tumor tissue group, but it was enhanced in BLCA (~5.2-fold), BRCA (~1.2-fold), HNSC (~2.8-fold), LUAD (~2.7-fold), SKCM (~6.8-fold), and UCEC (~4.1-fold), while no comparative non-tumor data was provided for glioblastoma (GBM), acute myeloid leukemia (LAML), ovarian carcinoma (OC), and skin cutaneous melanoma (SKCM), while no comparative non-tumor data was available for LAML and OV (Figure 1C). As with SOD2 expression, the hsa-miR-324-5p expression data for CRC in the PANCANCER cohort was consistent with data obtained from analysis of the TCGA CRC dataset (*n* = 325), with a markedly down-regulated hsa-miR-324-5p expression level in the CRC compared to the non-tumor colorectal tissues (~0.26-fold, *p* = 1.03 × 10^−13^) (Figure 1D). This finding, at least in part, is indicative of the tumor-promoting role of reciprocal down-regulation of SOD2 and hsa-miR-324-5p gene expressions in various human cancer types, including CRC cells.

### 2.2. SOD2 Interacts Directly with and Suppresses hsa-miR-324 Expression in CRC Cells 

**Figure 2:**
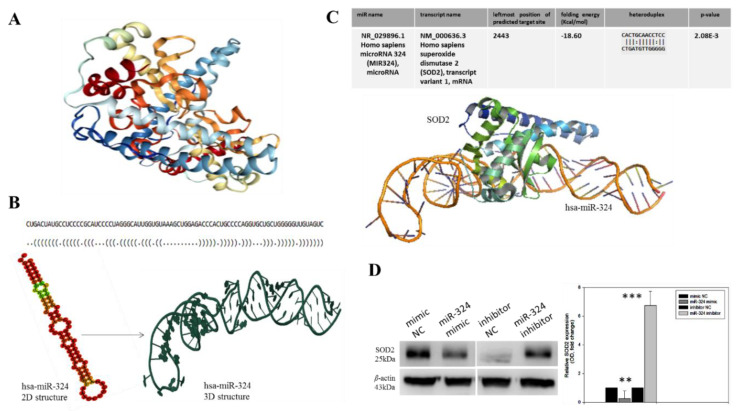
SOD2 interacts with, and is modulated by hsa-miR-324. (**A**) Crystal structure of the human manganese superoxide dismutase, PDB: 2ADQ. (**B**) The optimal secondary structure in dot-bracket notation with a minimum free energy (MFE) of −35.90 kcal/mol, (**upper panel**). The RNAfold-generated MFE secondary folding structure pattern of hsa-miR-324 (**lower panel, left**). The hsa-miR-342 tertiary structure generated based on the secondary structure in dot-bracket notation (**lower panel, right**). (**C**) Molecular docking showing the direct interaction between SOD2 and hsa-miR-324 with a shape complementarity/docking score of 14,236, Atomic contact energy (ACE) of −33.40 and an approximate SOD2-hsa-miR-324 complex interface area of 1896.30, using a root mean square distance (RMSD) of 9 for the final docking. The 3D transformation data, consisting of three rotational angles (38.44°, 20.02°, 216.62°) and three translational parameters (−0.02, 0.94, −1.95) applied on the ligand molecule, hsa-miR-324. (**D**) Representative Western blot image (**left panel**) and quantitative graphical representation (**right panel**) of the effect of variations in hsa-miR-324 expression on the expression level of SOD2 protein. β-actin served as loading control. NC, negative control; OD, optical density; * *p* < 0.05, ** *p* < 0.01, *** *p* < 0.001.

To better understand the relationship between SOD2 and hsa-miR-324-5p, as well as to rule out any modulatory loop between them in CRC cells, we employed a bioinformatics approach to generate the crystal structure of SOD2 based on PDB:2ADQ from the Protein Data Bank (www.rcsb.org/pdb/gene/SOD2; Figure 2A). For visualization of our hypothesized molecular interaction between hsa-miR-324-5p and SOD2, we generated the tertiary (3D) structure of hsa-miR-324-5p from its sequence-derived ‘brackets and dots’ linear structure and the minimum free energy (MFE) secondary (2D) structure (Figure 2B). We then used the EduPyMoL molecular interaction and visualization software to demonstrate the interaction between SOD2 and hsa-miR-324-5p, observing a high interaction propensity, broad conservation, and good complementarity between the 5′ end of hsa-miR-324-5p and the 3′ end of SOD2, demonstrated by a shape complementarity/docking score of 14,236, an atomic contact energy (ACE) of −33.40, a solvation folding energy of −18.60 Kcal/mol, an approximate SOD2-hsa-miR-324 complex interface area of 1896.30, when a root mean square distance (RMSD) of 9 is used for the final docking, and a *p*-value of 2.08 × 10^−3^ (Figure 2C). Moreover, to demonstrate that the observed SOD2-hsa-miR-324 interaction was modulatory in nature, we transfected the human CRC cell line DLD-1 with a hsa-miR-324 negative control (NC), mimic, or inhibitor. We observed significant down-regulation in the SOD2 protein expression following transfection of the DLD-1 cells with the hsa-miR-324 mimic, compared to the mimic NC-transfected cells (0.24-fold; *p* < 0.01). Conversely, transfection with hsa-miR-324 inhibitor elicited marked up-regulation of the SOD2 protein expression level, in comparison with the inhibitor NC-transfected cells (6.8-fold; *p* < 0.001) (Figure 2D). These findings indicated that SOD2 interacts directly with, and suppresses hsa-miR-324 expression in CRC cells.

### 2.3. Aberrant Expression of SOD2 Correlates with Disease Progression and SOD2-Induced Down-Regulation of hsa-miR-324 Facilitates Poor Prognosis

**Figure 3:**
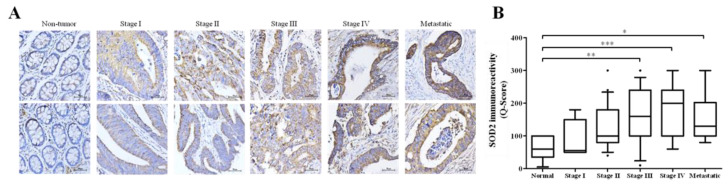

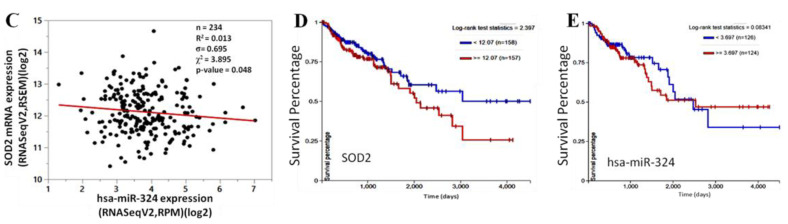
Aberrant expression of SOD2 correlates with disease progression, and SOD2-induced down-regulation of hsa-miR-324 facilitates poor prognosis. (**A**) Representative IHC images of differential SOD2 protein expression in non-tumor, stages I–IV and metastatic colon cancer tissue samples (*n* = 118). (**B**) Box and whiskers graph of the expression (Q-score) of SOD2 in stages I–IV and metastatic colon cancer tissue samples, compared to its expression in normal tissues. (**C**) A bivariate fit of SOD2 and hsa-miR-324 gene expression using data from the TCGA Colon Cancer (COAD) cohort dataset, TCGA. COAD. sampleMap/HiSeqV2, based on the gene-level transcription estimates, as in log2(*x* + 1) transformed RNA-Seq by Expectation–Maximization (RSEM) normalized count. Kaplan-Meier plots of the (**D**) SOD2 and (**E**) hsa-miR-324 differential expression-based overall survival rates of same TCGA Colon Cancer (COAD) cohort. * *p* < 0.05, ** *p* < 0.01, *** *p* < 0.001.

With an intention to establish a connection between the expression profiles of SOD2, hsa-miR-324 and disease progression in CRC patients, we probed for and analyzed probable correlations between the expression levels of SOD2 in stage I to stage IV, and metastatic CRC patients from the Taipei Medical University-Shuang Ho Hospital CRC cohort (*n* = 118). Our immunohistochemical (IHC) staining data demonstrated that relative to its expression in the normal colorectal tissue samples, the expression of SOD2 in the CRC tissues was significantly increased as the disease progressed from stage I to stage IV (stage II: 2.2-fold, *p* < 0.05; stage III: 3.1-fold, *p* < 0.01; stage IV: 4.4-fold, *p* < 0.001); similarly, an estimated 2.4-fold increase was observed in SOD2 expression in the metastatic disease group, compared to the normal group (*p* < 0.05) (Figure 3A,B). Having established a positive correlation between SOD2 expression, disease progression, and metastasis (Figure 3A,B), as well as corroborating the inverse correlation between SOD2 and hsa-miR-324 expression in CRC by using the TCGA colon cancer (COAD) dataset (*n* = 234, *σ*= 0.695, *p* = 0.048) (Figure 3C), we then evaluated the prognostic potential of SOD2 (*n* = 315) and hsa-miR-324-5p (*n* = 250) in CRC patients based on the TCGA COAD cohort using the Kaplan-Meier (KM) survival plot. Dichotomization of gene expression into high or low in the cohort was based on the median value of hsa-miR-324-5p or SOD2 expression. Patients with low SOD2 expression (<12.07; *n* = 158; 5-year overall survival (OS) rate = 63.50%; 10-year OS rate = 51.0%) exhibited a 7.25–26.0% (1.1–2.0-fold) survival advantage over the high SOD2 expression group (≥12.07; *n* = 157; 5-year OS rate = 56.25%; 10-year OS rate = 25.0%) (Figure 3D). In parallel analyses, patients with high hsa-miR-324-5p expression (≥3.70; *n* = 124) exhibited 5- and 10-year OS rates of 52.5% and 43.75% respectively, while for the low hsa-miR-324-5p group (<3.70; *n* = 126), the 5- and 10-year OS rates were 68.75% and 36.5%, revealing a time-dependent influence of hsa-miR-324-5p on the OS of CRC patients. Taken together, those findings do indicate that aberrant expression of SOD2 correlates with disease progression, and the SOD2-induced down-regulation of hsa-miR-324 facilitates poor prognosis, while suggesting the molecular targetability of SOD2 and potential therapeutic utility of hsa-miR-324-5p in advanced stage and metastatic CRC patients.

### 2.4. 4-AAQB Inhibits the Viability and/or Proliferation of Wild Type and Side-Population Human Colorectal Carcinoma Cells

**Figure 4:**
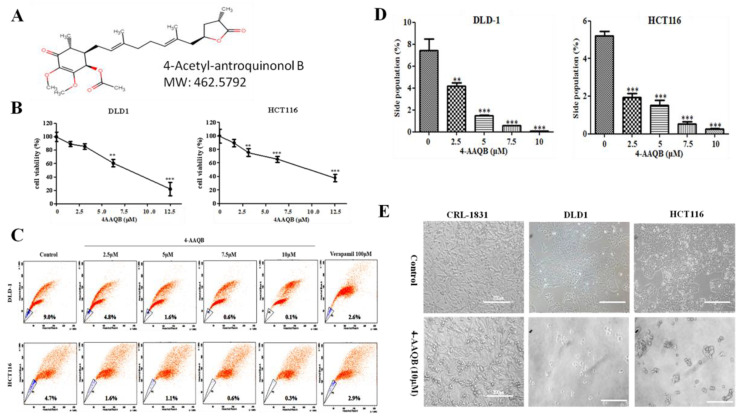
4-acetylantroquinonol B (4-AAQB) inhibits the viability and/or proliferation of human colorectal carcinoma side population cells. (**A**) The chemical structure of 4-acetylantroquinonol B (C_26_H_38_O_7_) with a molecular weight of 462.6 g/mol. (**B**) The inhibitory effect of 4-AAQB on the viability of DLD-1 (**left panel**) and HCT116 (**right panel**) colorectal cancer cell lines, using the sulforhodamine B (SRB) cytotoxicity assay. (**C**) Representative flow-cytometry dot plots of the dose-dependent attenuation of DLD-1 (**upper panel**) and HCT116 (**lower panel**) colorectal cancer side population cells by 4-AAQB, quantified by fluorescence-activated cell sorting (FACS) analysis. Control, cells were incubated in Hoechst 33342 alone (serving as negative control); Verapamil, cells with Hoechst 33342 accumulation in the presence of 100 μM verapamil (serving as positive control); 4-AAQB, cells with Hoechst 33342 accumulation in the presence of 2.5 μM–10 μM 4-AAQB. (**D**) Graphical representation of C. (**E**) Photomicrograph showing the morphological and quantitative changes in cultured 4-AAQB-treated CRL-1831 non-tumor colon cells, DLD-1 and HCT116 colorectal cancer side-population (SP) cells, compared to their untreated control counterparts.

Furthermore, we investigated the pharmacological targetability of the SOD2-enriched CRC cells, by evaluating the effect of 4-AAQB (Figure 4A) on the viability and proliferation of the CRC wild type or sorted side-population (SP) cells, against the background of 4-AAQB bioinformatics-based predicted health benefits in several human systems, with highest (~99%) health effects in the gastrointestinal system, which includes the colon and rectum (Supplementary Figure S1). Treatment with 2.5–12.5 μM 4-AAQB significantly suppressed the cell viability of DLD-1 or HCT116 cells in a dose-dependent manner, and with 24 h IC_50_ of 7.82 μM or 11.25 μM, respectively (Figure 4B). More importantly, considering the clinical relevance of CSC targeting in effective anticancer therapies, we demonstrated that exposure to 2.5 μM, 5 μM, 7.5 μM, or 10 μM 4-AAQB reduced the proportion of DLD-1 SP cells by 4.2% (*p* < 0.01), 7.4% (*p* < 0.001), 8.4% (*p* < 0.001), or 8.9% (*p* < 0.001), compared to 6.4% by 100 μM verapamil, which specifically inhibits the Hoechst dye-extruding potential of the ATP-binding cassette (ABC) membrane transporter. For the HCT116 SP cells treated with 2.5 μM, 5 μM, 7.5 μM, or 10 μM 4-AAQB, we observed 3.1% (*p* < 0.001), 3.6% (*p* < 0.001), 4.1% (*p* < 0.001), or 4.4% (*p* < 0.001) reduction, respectively, in comparison with 1.8% by 100 μM verapamil (Figure 4C). Additionally, we observed a marked reduction in the SP cell number (Figure 4D), as well as significant cell death and dysmorphism in cultured DLD-1 or HCT116 SP cells exposed to 10 μM 4-AAQB compared to the untreated control group; interestingly, 4-AAQB exhibited no significant inhibitory effect on the viability and/or proliferation of the non-tumor colon cell line, CRL-1831 (Figure 4E). This data indicates that 4-AAQB inhibits the viability and/or proliferation of human colorectal carcinoma wide-type and side-population cells, as well as corroborating the findings of our previously published work [8].

### 2.5. The Anticancer Effect of 4-AAQB, Akin to SOD2-Silencing, Is Mediated by hsa-miR-324 and Associated with Epithelial-to-Mesenchymal Transition (EMT)- and Cell-Death-Related Molecular Changes in Colorectal Cancer SP Cells

**Figure 5:**
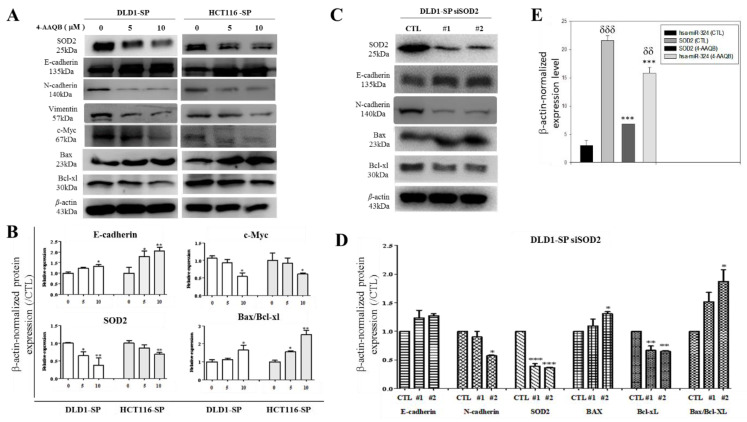
The anticancer effect of 4-AAQB is mediated by hsa-miR-324 and this is consistent with EMT- and cell-death-related molecular changes in colorectal cancer SP cells. (**A**) 4-AAQB down-regulates N-cadherin, vimentin, C-myc, SOD2, and Bcl-xl, but up-regulates E-cadherin and Bax. (**B**) Semi-quantitative analyses of E-cadherin, N-cadherin, SOD2, and the ratio of Bax and Bcl-xl protein expression in DLD1 and HCT116 cells treated with 4-AAQB. (**C**) SOD2 small interfering RNA (siRNA) knockdown DLD1 shows down-regulation of N-cadherin, with up-regulation of E-cadherin and Bax. (**D**) Semi-quantitative analyses of E-cadherin, N-cadherin, SOD2, Bax, Bcl-xl, and the ratio of Bax and Bcl-xl transcript expression in the siSOD2 DLD1 cell line by RT-PCR. * *p* < 0.05, ** *p* < 0.01, *** *p* < 0.001. (**E**) Graphical representation of the effect of 10 μM 4-AAQB treatment on SOD2 mRNA and hsa-miR-324 expression levels in DLD1 SP cells evaluated by RT-PCR. Statistical significance is indicated using * for CTL vs. 4-AAQB, and δ for hsa-miR-324 vs. SOD2. *δδp* < 0.01, *** *p* < 0.001; SP, side population cells; #1, siSOD2 clone 1; #2, siSOD2 clone 2.

Investigating the molecular mechanism underlying the observed inhibitory effect of 4-AAQB in the SOD2-rich CRC SP cells, which serve as in vitro CRC-SC models, the results of our Western blot analyses demonstrate that exposure to 5–10 μM 4-AAQB significantly down-regulates the expression level of SOD2, N-cadherin, vimentin, c-Myc, and Bcl-xL proteins, while up-regulating the expression of E-cadherin and Bax in the DLD-1 or HCT116 SP cells (Figure 5A,B). Similarly, in parallel assays, transient transfection of the DLD-1 SP cells with short interfering RNA specifically targeting SOD2 (siSOD2) resulted in the co-suppression of SOD2, N-cadherin, and Bcl-xL protein expression, while conversely enhancing E-cadherin and Bax protein expression levels (Figure 5C). Consistent with Figure 5C, the semi-quantitative analyses of E-cadherin, N-cadherin, SOD2, Bax, and Bcl-xl protein expression, as well as the Bax/Bcl-xL ratio in the DLD-1 siSOD2 cell line by RT-PCR exhibited similar expression profiles (Figure 5D). Both the 4-AAQB and siSOD2-mediated proteomic and transcriptomic profiles revealed an increase in the Bax/Bcl-xL ratio. Furthermore, using the RT-PCR, we demonstrated that DLD-1 SP cells treated with 10 μM 4-AAQB exhibited significantly suppressed SOD2 mRNA expression, and conversely up-regulated hsa-miR-324-5p expression. These data suggest that the anticancer effect of 4-AAQB, akin to SOD2-silencing, is mediated by hsa-miR-324 and it is associated with EMT- and cell-death-related molecular changes in the colorectal cancer SP cells, at both the transcript and protein levels. 

### 2.6. hsa-miR-324-Inducing 4-AAQB Inhibits SOD2-Mediated Motility, Invasiveness and Clonogenicity of Colorectal Cancer SP Cells

**Figure 6:**
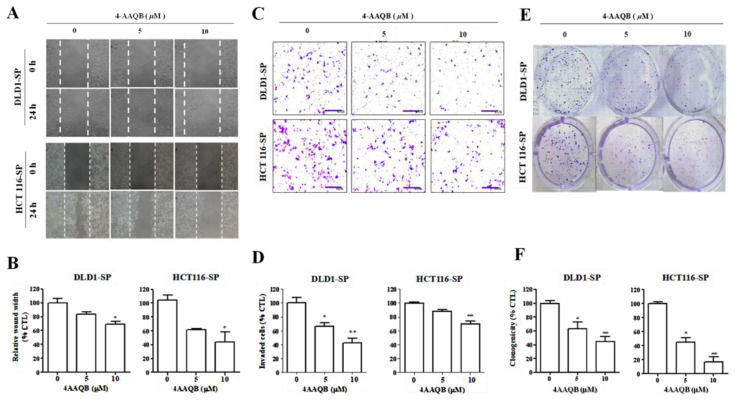
hsa-miR-324-inducing 4-AAQB inhibits the SOD2-mediated motility, invasiveness, and clonogenicity of colorectal cancer SP cells. (**A**) Representative micrograph showing the dose-dependent effect of 4-AAQB on the migration of DLD1 and HCT116 SP cells at the 24 h time-point, using the scratch-wound healing assays. (**B**) Histographs of the estimated wound closure or migration by DLD1 and HCT116 colorectal cancer SP cells exposed to 5 μM or 10 μM 4-AAQB, compared with the untreated control SP cells. (**C**) Micrographs and (**D**) histograms showing the dose-dependent reduction in number of invaded SP cells, after 4-AAQB treatment of DLD1 and HCT116 SP cells, as evaluated by the matrigel invasion assay. (**E**,**F**) Dose-dependent attenuation of clonogenicity after 4-AAQB treatment of DLD1 and HCT116 SP cells. Data are expressed as the mean ± S.E.M. and are representative of experiments performed thrice in triplicate. * *p* < 0.05, ** *p* < 0.01, *** *p* < 0.001.

Having established that 4-AAQB effectively elicits the up-regulation of hsa-miR-324 while down-regulating SOD2 expression, we further investigated the effect of 4-AAQB treatment on the motility and oncogenicity of CRC-SCs using the sorted DLD-1 or HCT116 SP cells. We demonstrated that compared to the observed migration by the untreated cells, exposure to 5–10 μM 4-AAQB significantly attenuates the ability of the SP cells to migrate in a dose-dependent manner over a 24 h time-course (Figure 6A). At the 24 h time-point, the DLD-1 SP cells treated with 5–10 μM 4-AAQB had lost ~20–29% migratory potential, while the HCT116 SP cells recorded a 41–59% lag in migration, in comparison to observed migration by their untreated counterparts (Figure 6B). Similarly, 4-AAQB treatment elicited a significant reduction in the number of invaded DLD-1 SP (5 μM: 35%, *p* < 0.05; 10 μM: 60%, *p* < 0.01) or HCT116 SP (5 μM: 11%, *p* = non-significant; 10 μM: 31%, *p* < 0.01) cells (Figure 6C,D). Moreover, we observed that there was a significantly attenuated capacity to form colonies in the 4-AAQB-treated SP cells, in comparison to the untreated SP cells (Figure 6E,F). These results indicate that 4-AAQB effectively inhibits the SOD2-mediated motility, invasiveness, and the clonogenicity of CRC-SC cells. 

### 2.7. 4-AAQB Suppresses the Cancer Stem Cell-Like Phenotype of Colorectal Cancer Cells

**Figure 7:**
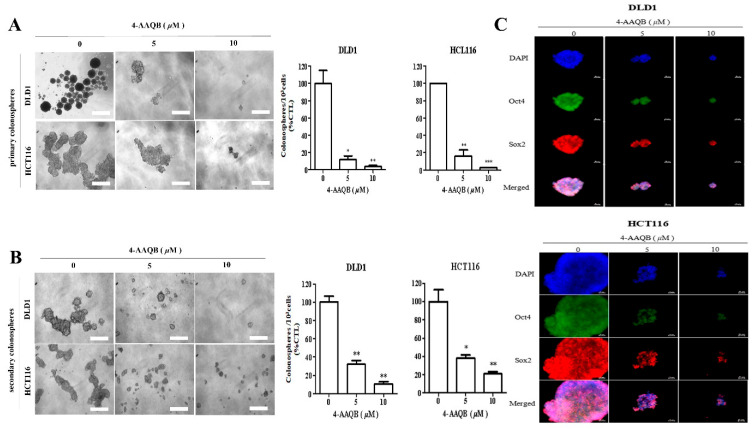
4-AAQB suppresses the cancer stem cell-like phenotype of colorectal cancer cells. (**A**) DLD1 and HCT116 cells pre-treated with 5 or 10 µM 4-AAQB for 48 h exhibit a decreased colonosphere size (**left panel**) and number (**right panel**) in both primary (**A**) and secondary (**B**) generation tumorspheres. (**C**) Treatment with 4-AAQB attenuated OCT4 and SOX2 immunoreactivity and inhibited their nuclear co-localization in DLD1- or HCT116-derived colonospheres using the immunofluorescent (IFC) staining technique. Data are expressed as the mean ± S.E.M. and are representative of experiments performed in triplicate. * *p* < 0.05, ** *p* < 0.01, *** *p* < 0.001 compared with untreated control.

In parallel assays to confirm the inhibitory effect of 4-AAQB on CRC-SCs, after formation of primary colonospheres by wild-type DLD-1 or HCT116 cells cultured in stem cell media, we demonstrated marked dose-dependent attenuation of the viability and proliferative ability of the formed primary DLD-1 or HCT116 colonospheres when treated with 5 μM (DLD-1: 88% attenuation, *p* < 0.05; HCT116: 84% attenuation, *p* < 0.01), or 10 μM (DLD-1: 97% attenuation, *p* < 0.05; HCT116: 98.6% attenuation, *p* < 0.001) of 4-AAQB (Figure 7A). Since at the core of CRC cell resistance to chemotherapeutics, metastatic disease progression, and tumor recurrence lies the characteristic CSC ability to regenerate all facets of the original/primary tumor and drive malignantization [19,20], we examined if and how 4-AAQB affects the colonosphere-forming ability of single cell solutions derived from dissociated primary colonospheres and cultured in stem cell media (recapitulating CRC-SC self-renewal) in the presence of 5–10 μM 4-AAQB. We demonstrated that 4-AAQB significantly inhibited the self-renewal capacity of the DLD-1 (5 μM: 69% inhibition, *p* < 0.01; 10 μM: 90% inhibition, *p* < 0.01) or HCT116 (5 μM: 61% inhibition, *p* < 0.05; 10 μM: 80% inhibition, *p* < 0.01) primary colonospheres (Figure 7B). We further demonstrated that the quantitative and qualitative attenuation of DLD-1 or HCT116 colonosphere formation positively correlated with pronounced suppression of the nuclear expression of the pluripotency transcription factors, OCT4 and SOX2, in a dose-dependent manner (Figure 7C). These data indicate that 4-AAQB efficaciously suppresses the CSC-like phenotype, including the proliferation and self-renewal of CRC cells, in vitro.

### 2.8. 4-AAQB Attenuate Resistance to FOLFOX and Reduce the Tumorigenicity of Colorectal Cancer Cells by Suppressing SOD2 and Up-Regulating hsa-miR-324-5p Expression, In Vivo 

**Figure 8:**
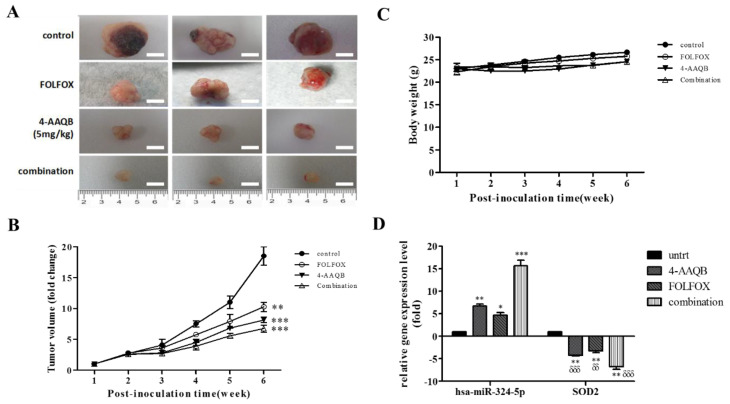
4-AAQB alone or in synergism with FOLFOX (folinate (leucovorin), fluorouracil (5FU), and oxaliplatin) reduces the tumorigenicity of colorectal cancer cells by suppressing SOD2 and up-regulating hsa-miR-324-5p expression, in vivo. (**A**) Colorectal cancer cell line, DLD1 cells were injected into the right flank of the animals at a concentration of 1 × 10^5^ cells/100 μL phosphate buffered saline (PBS). One week after tumor injection, different treatment regimens were commenced. Photographs of tumor-bearing mice are shown (*n* = 5 each group) with treatment regimens labelled on the left-hand column. (**B**) Graphical representation of treatment-related fold-change in tumor volume over time. The tumor volume of each group was measured each week using a caliper. Treatment started a week after tumor xenografting and lasted 5 weeks. (**C**) Correlation between different treatment regimens and cancer-associated body weight change. (**D**) Graphical representation of the effect of FOLFOX and/or 4-AAQB on SOD2 mRNA and hsa-miR-324 expression levels in the resected tumor tissue from treated mice (*n* = 5/each treatment regimen), compared to untreated group (*n* = 5), as evaluated by RT-PCR. Statistical significance is indicated using * for untreated vs. treated, and ^δ^ for hsa-miR-324-5p vs. SOD2. *^δδ^ p* < 0.01, *^δδδ^ p* < 0.001, * *p* < 0.05, ** *p* < 0.01, *** *p* < 0.001. untrt, untreated control.

Since our team is practice-oriented, we pre-clinically probed for the translational relevance of our in vitro findings by assessing the in vivo effect of 4-AAQB and/or FOLFOX (folinate (leucovorin), fluorouracil (5FU), and oxaliplatin) on the growth of CRC cells in the BALB/c mice (average body weight, 22.5 ± 3.74 g) xenograft model. Results of our tumor xenograft assays showed that by end of week 6, the tumors grown in mice inoculated with untreated DLD-1 cells (*n* = 5) were numerically more and much larger than those in mice treated with FOLFOX (*n* = 5, *p* < 0.01), 4-AAQB (*n* = 5, *p* < 0.001) or a combination of 4-AAQB and FOLFOX (*n* = 5, *p* < 0.001), in descending order of degree (Figure 8A,B). Macro-anatomically, the average weights of the tumors that were resected from the 4-AAQB- and combination-treated mice were significantly lesser than that of the FOLFOX- treated or untreated control mice (4-AAQB vs. control: 334.3  ±  79.4 mg vs. 3674.1  ±  593.1  mg, *p*  <  0.001; Combination vs. control: 282.6  ±  46.8 mg vs. 3674.1  ±  593.1 mg, *p*  <  0.001; FOLFOX vs. control: 459.3  ±  57.9 mg vs. 3674.1  ±  593.1 mg, *p*  <  0.01) (Figure 8A,B), indicating that the xenograft tumor growth was significantly inhibited by 4-AAQB alone or in combination with FOLFOX, compared with the FOLFOX- treated or untreated group. Additionally, while the mean body weight of 4-AAQB- or dual agent- treated tumor-inoculated mice was stable, we observed a slight increase (~2 g) in those treated with FOLFOX, as well as in the untreated control group (~4 g) by end of week 6 (Figure 8C). The observed increase in body mass in the untreated and FOLFOX-treated littermates were however, statistically insignificant, and all mice (*n* = 5/treatment regimen) exhibited normal feeding habits and motor activity. Finally, and interestingly, we demonstrated robust reciprocity in the differential expression of SOD2 and hsa-miR-324-5p in the tumors resected from the xenograft mice in all treatment groups, using RT-PCR (Figure 8D). We observed statistically significant moderate (*p* < 0.01), mild (*p* < 0.05), or strong (*p* < 0.001) increase in the expression of hsa-miR-324-5p in mice treated with 4-AAQB alone, FOLFOX alone, or 4-AAQB + FOLFOX combination, respectively (Figure 8D). Those results indicate that 4-AAQB is non-toxic to non-cancerous tissues (also see Supplementary Figure S2), and that alone or in synergism with FOLFOX, 4-AAQB significantly inhibits CRC xenograft tumor growth in vivo.

## 3. Discussion

In our previous work [8] on the role of 4-AAQB in CRC, we provided evidence that 4-AAQB exhibits potent antiproliferative and anti-CSC therapeutic effects in CRC. We showed that 4-AAQB effectively reverses or attenuates the resistance of cancer cells to 5-FU or FOLFOX anticancer therapy, thus, enhancing chemosensitivity both in vitro and in vivo. Furthermore, we demonstrated that the JAK/STAT signaling pathway plays a vital role in the 4-AAQB-mediated suppression of CRC-SCs, thus projecting it as a putative therapeutic target in CRC treatment. Thus, we concluded that the relatively novel phytoalexin, 4-AAQB, was a potent therapeutic agent for single-agent or components of standard combination chemotherapy. With increased understanding of the epigenetic landscape in cancer pathologies, there has been increased interest and investigation of an epigenetics-mediated multipronged targeted-therapy strategy in tackling the clinical challenge of primary or evolved required resistance [21,22,23]. 

In this present study, consistent with current knowledge that the epigenetic modulation of genes is a critical mechanism underlying the CRC oncogenicity and disease progression, as well as the development of resistance to chemotherapeutics, we demonstrate a novel and previously undocumented epigenetic-based mechanism of 4-AAQB therapeutic activity in CRC, involving the down-regulation of tumor-promoting SOD2 by the suppressor ncRNA, hsa-miR-324-5p. This negative epigenetic modulation of SOD2 was shown to be associated with the converse enrichment of hsa-miR-324 and through the interaction of the 3′-UTR of SOD2 mRNA to the 5′-UTR of hsa-miR-324 (Figure 1 and Figure 2). Located on chromosome 6 (6q25.3 region) and resident in the mitochondrial matrix, the human homotetrameric SOD2 consists of four Mn^3+^-harboring sub-units, catalyzes the disproportionation of free radical superoxides to O_2_ and H_2_O_2_, and lowers cell susceptibility to oxidative injury in a genotoxic condition [12,13]. In most of the early documentations, SOD2 was implicated as a tumor suppressor [24]; however, more recently, there is accruing evidence that of the oncogenic/tumor-promoting role of SOD2 [13,14,15]. Consistent with the latter, in this study we demonstrated the oncogenic role of the aberrant expression of SOD2 in CRC, as evidenced by its ability to facilitate metastatic disease progression through the repression of hsa-miR-324 (Figure 3). In addition, we also showed that 4-AAQB inhibits the viability and/or proliferation of human CRC SP cells in a hsa-miR-324-mediated manner, with associated attenuation of the SOD2-facilitated EMT and enhanced cell-death in the SP cells (Figure 4 and Figure 5; Supplementary Figure S1). These findings are consistent with the demonstrated suppression of the invasion and migration of hepatocellular carcinoma (HCC) cells by hsa-miR-324-5p-induced down-regulation of the specificity protein 1 (SP1) and E26 transformation-specific protein 1 (ETS1) [25], where decreased SP1 binding to the SOD2 promoter inhibits the constitutive activation of SOD2 in breast cancer cells [13], since the proximal promoter that mediates the transcription of SOD2 is TATA-less and contains binding sites for several transcription factors, including ETS1 and SP1. It is of therapeutic relevance that the hsa-miR-324-inducing 4-AAQB inhibits the SOD2-mediated motility, invasiveness, and clonogenicity of colorectal cancer SP cells (Figure 6).

Like most malignancies, CRC is a SC-driven pathology, characterized by the presence of a sub- or side-population within the tumor bulk which is characteristically quiescent, phenotypically *ALDH*+, functionally capable of self-renewal, and underlies innate or acquired insensitivity to chemotherapy, metastasis, and disease recurrence [26,27]. The targeting and killing of these CSC-like SP cells by 4-AAQB is posited as a rational and efficacious therapeutic approach, as it eliminates the quiescent, slowly-dividing, and characteristically therapy-resistant tumor-initiating (and maintaining-) cells, alongside the sensitive rapidly-dividing non-SP cells. Consistent with this, we provided evidence that 4-AAQB, in a dose-dependent manner, effectively targets the primary colonospheres and subsequent generations of colonospheres, recapitulating the pharmacological inhibition of CRC-SC-related self-renewal or propagation, with associated attenuation of the expression of critical pluripotency transcription factors (Figure 7). 

Understanding that self-renewal, a vital property of the CSC-like SP cells, is associated with the facilitation and maintenance of the proliferative capacity of cancerous cells, makes the clinical implication of our data apparent, as it highlights the putative ability of 4-AAQB to negatively modulate oncogenic self-renewal signaling, thus enhancing the sensitivity of malignant cells to chemotherapeutics, altering their survival strategies, limiting tumorigenesis and/or oncogenicity, and apparently impeding disease recurrence [28]. From in vivo validation of our in vitro findings, we confirmed that 4-AAQB alone or in synergism with FOLFOX reduces the tumorigenicity of CRC cells by suppressing SOD and up-regulating hsa-miR-324 expression, in vivo (Figure 8). This is clinically relevant and important, not only because it is, to the best of our knowledge, the first demonstration of an hsa-miR-324-5p-mediated anticancer activity of 4-AAQB in CRC, but also because despite advances in anticancer therapy, resistance to chemotherapy remains a great challenge in the long-term management of incurable metastatic disease, and it subsequently contributes to cancer-related mortality. FOLFOX is the therapeutic mainstay in postoperative CRC patients, having been shown in several trials to contribute to increased progression-free (PFS) and overall (OS) survival, with response rates as high as 50%; however, resistance to FOLFOX does occur because of its reduced intracellular entry or enhanced efflux from the CRC cells by ABC-type multi-drug resistance (MDR) transporters or copper-transporting p-type ATPases (reviewed in [29]). In the light of this, we showed in addition that 4-AAQB enhances the sensitivity of CRC cells to the standard of care FOLFOX, and significantly potentiates the anticancer effect of FOLFOX in the murine CRC xenograft models (Figure 8; Supplementary Figure S2). 

Ranking as the third most common cause of cancer and the fourth most common cause of cancer-related deaths worldwide, CRC remains a major health problem. FOLFOX, comprising of folinate (leucovorin), fluorouracil (5FU), and oxaliplatin, is currently the drug of choice for patients with CRC; however, some patients are non-responsive or develop insensitivity to FOLFOX, resulting in treatment failure and consequently, tumor progression, thus, necessitating a therapeutic approach with enhanced efficacy. This present study provides evidence that 4-AAQB alone or by synergistically interacting with FOLFOX, exhibits an enhanced ability to kill CRC cells, inducing marked apoptosis via an epigenetically modulated pathway. Mechanistically, hsa-miR-324-mediated inhibition of the SOD2-activated oncogenicity and the cancer stem cell-like phenotype of CRC cells is a probable critical component of our observed 4-AAQB—FOLFOX anticancer synergism.

Interestingly, corroborating the critical role of epigenetic alterations in the unrestrained growth, invasion, colonization and acquired resistance of cancerous cells [16,17,18,30,31], we validated the reciprocal suppression of the pro-tumorigenic and pro-metastatic SOD2 and suppressor miR, hsa-miR-324 ex-vivo, showing that the expression of hsa-miR-324 levels in the resected tumor tissue from 4-AAQB- and/or FOLFOX- treated mice was significantly up-regulated, while conversely, that of SOD2 was reduced markedly, compared to the untreated group (Figure 8). Overall, the data demonstrates that the hsa-miR-324/SOD2 signaling axis is a putative novel therapeutic target, and a potential prognostic marker in patients with CRC.

**Scheme 1:**
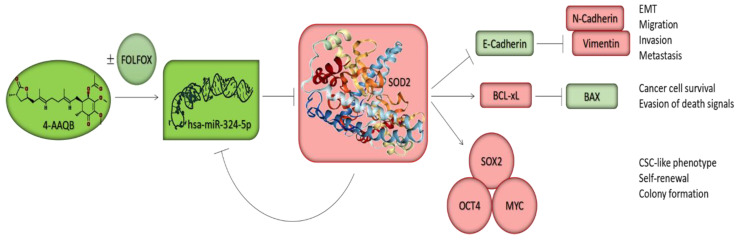
A pictorial summary of the anticancer therapeutic activity of 4-AAQB, alone or in combination with FOLFOX against CSCs in CRC cells. 4-AAQB inhibits the aberrant expression of SOD2 through the re-expression of hsa-miR-324-5p, the negative modulation of pluripotency transcription factors, the augmentation of the BAX/BCL-xL ratio, and the reprogramming of malignant colorectal cancer cells from the aggressive mesenchymal phenotype to a relatively benign epithelial phenotype, subsequently enhancing the sensitivity of the cancer cells to conventional chemotherapy and facilitating better prognosis.

Put together, this present study uncovers a novel mechanistic underlining the therapeutic effect of 4-AAQB alone or in combination with FOLFOX in the activation of cell death signals, attenuation of CSC-like phenotypes, and induction of apoptosis in the CRC cells. Akin to the single-agent therapy, the pharmacological synergism involves multiple mechanisms as summarized in the Scheme 1. The enhanced cytotoxicity of 4-AAQB—FOLFOX dual-agent therapy is clinically relevant. We propose that SOD2-enrichment in the CRC cells accentuates mitochondrial biogenesis, activates the intracellular anti-oxidative machinery, alleviates oxidative stress, and enhances the maintenance of the CSC-like phenotype, as well as acquisition of resistance to chemotherapeutic agents, by functional suppression of hsa-miR-324-5p expression and/or activity; however, exposure to 4-AAQB induces the re-expression of hsa-miR-324-5p, attenuates SOD2 expression, and sensitizes the CSC-like SP cells in CRC to FOLFOX therapy. Thus, we propound a novel perspective on the putative roles of 4-AAQB as a hsa-miR-324-mediated CSC-targeting small molecule inhibitor of SOD2 in CRC, in vitro and in vivo.

## 4. Materials and Methods

### 4.1. Reagents and Drugs

The compound 4-acetylantroquinonol B (4-AAQB, >99% purity) was obtained from New Bellus Enterprises Co., Ltd. (Tainan, Taiwan), while Leucovorin calcium (PHR1541 SIGMA-ALDRICH, 5-FU (F6627 SIGMA, ≥99% HPLC) and oxaliplatin (PHR1528 SIGMA-ALDRICH) were purchased from Sigma-Aldrich, Inc. (St. Louis, MO, USA). Stock solutions of 10 mM in dimethyl sulfoxide (DMSO, Sigma-Aldrich) or sterile ddH_2_O for 4-AAQB or FOLFOX, were stored at −20 °C or 4 °C, respectively, until use. Anti-SOD2 (ab13533 Rabbit pAb) antibody was purchased from Abcam plc. (Biochiefdom International Co., Ltd., New Taipei City, Taiwan), antibodies against E-cadherin (24E10: #3195, Rabbit mAb), N-cadherin (D4R1H: #13116, Rabbit mAb), and vimentin (D21H3: #5741, Rabbit mAb) were purchased from Cell Signaling Technology (CST, Beverly, MA, USA). Anti-c-Myc (9E10: sc-40), Oct4 (C-10: sc-5279), Sox2 (E-4: sc-365823), Bax (B-9: sc-7480), Bcl-xL (7B2.5: sc-56021), and β-actin (C4: sc-47778) antibodies were purchased from Santa Cruz Biotechnology (Santa Cruz, CA, USA). Alexa Fluor 647 donkey anti-rabbit IgG and Alexa Fluor 488 donkey anti-rabbit IgG were purchased from Invitrogen (Grand Island, NY, USA).

### 4.2. Cells and Cell Culture

The human non-tumor colon epithelial cell line FHC (ATCC-CRL-1831), as well as the CRC cell lines DLD-1(ATCC-CCL-221) and HCT116 (ATCC-CCL-247) were purchased from the American Type Culture Collection (ATCC, Manassas, VA, USA), and cultured in DMEM/F-12 (Gibco^TM^, 12-634-010; Thermo Fisher Scientific Inc., Waltham, MA, USA), RPMI-1640 (Gibco^TM^, 11-875-119) and McCoy’s 5A modified (Gibco^TM^, 16-600-082) medium, respectively. Both media were supplemented with 10% fetal bovine serum (FBS, Sigma, F7524) and 1% penicillin-streptomycin (Gibco^TM^, 15140) at 37 °C, in a 5% humidified CO_2_ incubator (Shel Lab, Sheldon Manufacturing Inc, Cornelius, OR, USA). Cells were sub-cultured at 90% confluence and the media changed every 72 h.

### 4.3. Access and Probe of Online Cancer Data Set

The Cancer Genome Atlas (TCGA) PANCANCER cohort dataset consisting of 14 cancer types were accessed, and analyses of the expression profile of SOD2 and hsa-miR-324 in normal–cancer tissue pairs was performed using the StarBase v2.0 software algorithms [30]. The TCGA colon cancer (COAD) cohort was also analyzed for the influence of SOD2 or hsa-miR-324 expression on survival rates.

### 4.4. miRNA Profiling and Secondary Structure Prediction

We used TargetScan (http://www.targetscan.org/), and miRDB (http://www.mirdb.org/) for miR profiling and target sorting. The M-FOLD program v 2.3 The M-FOLD program (http://mfold.rna.albany.edu/?q=mfold) was used to predict the secondary structure of hsa-miR-324. The prediction was done as described earlier by Bellucci and colleagues [31].

### 4.5. Cell Viability and Drug Combination Assays 

CRC cells were seeded in supplemented media at a density of 4 × 10^3^ cells/well in triplicates in 96-well plates and incubated for 24 h in humidified 5% CO_2_ at 37 °C before exposure to different concentrations of 4-AAQB for 48 h. Cell viability and/or proliferation were assessed by a sulforhodamine B (SRB, Sigma; 59012-5G) colorimetric assay following the manufacturer’s instructions. Untreated wild type cells served as control. The assay was performed three times in triplicates. Optical density (OD) was measured at 495 nm wavelength, using a SpectraMax microplate reader (Molecular devices, Kim Forest Enterprises Co., Ltd., New Taipei City, Taiwan). 

### 4.6. Flow Cytometry-Based Side Population Analysis

To identify the CRC side population (SP), the DLD-1 or HCT116 cells were washed in warm RPMI-1640 media supplemented with 2% FBS and 10 mmol/L HEPES [4-(2-hydroxyethyl)-1-piperazineethanesulfonic acid] buffer (Invitrogen-Life Technologies, Carlsbad, CA, USA), re-suspended at a density of 1 × 10^6^ cells per mL of RPMI-1640 supplemented with 2% FBS and 10 mmol/L HEPES buffer containing 5 μg/mL Hoechst 33342 dye, and incubated at 37 °C for 1.5 h with gentle agitation. As control, SP cells were incubated with 50 μM verapamil (Sigma-Aldrich), an ABC transporter inhibitor, washed with ice-cold Hank’s balanced salt solution (HBSS) supplemented with 2% FBS and 10 mmol/L HEPES buffer, centrifuged, and re-suspended in ice-cold supplemented HBSS. Cells were exposed to 1 μg/mL 7-Aminoactinomicin D (7-AAD, #A1310, Molecular Probes, Thermo Fisher Scientific Inc., Eugene, OR, USA) to delineate only viable cells. To obtain a single cell suspension, cells were filtered through a 70-μm filter, sorted into SP and non-SP cell fractions, then analyzed on the BD FACSAria II System (BD Biosciences, San Jose, CA, USA). The purity ≥ 98%.

### 4.7. Western Blot Analysis

Cultured CRC cells were harvested and washed thrice with PBS and lysate prepared using ice-cold lysis buffer solution. After being heated at 95 °C for 5 min, immunoblotting of the protein lysate was performed. Blots were blocked for 1 h with 5% skimmed milk in Tris Buffered Saline with Tween 20 (TBST), incubated overnight at 4 °C with specific primary antibodies against SOD2 (1:1000), E-cadherin (1:2000), N-cadherin (1:2000), vimentin (1:1000), c-Myc (1:1000), BAX (1:1000), BCL-xL (1:1000), and β-actin (1:500). Thereafter, the polyvinylidene difluoride (PVDF) membranes were washed three times with TBST, then incubated with horseradish peroxidase (HRP)-labeled secondary antibody for 1 h at room temperature and washed with TBST again before band detection using enhanced chemiluminescence (ECL) Western blotting reagents and imaging with the BioSpectrum Imaging System (UVP, Upland, CA, USA).

### 4.8. miRNA and Small Interfering RNA (siRNA) Transfection

For miRNA transfection, micrON^TM^ hsa-miR-324-5p mimic (#miR10000761-1-5), micrOFF^TM^ hsa-miR-324-5p inhibitor (#miR20000761-1-5), micrON^TM^ mimic negative control #22 (# miR01101-1-5) and micrOFF^TM^ inhibitor negative control #22 (#miR02101-1-5) were purchased from RiboBio (Guangzhou RiboBio Co. Ltd, Guangzhou, China). For transient silencing of SOD2, the SOD2-specific siRNA (si-h-SOD2_001, B000006648A-1-5) were also purchased from RiboBio (Guangzhou RiboBio Co. Ltd., Guangzhou, China). Lipofectamine 2000 (Invitrogen, Carlsbad, CA, USA) was used for the transfection of the miRNA oligonucleotides and siRNAs following the manufacturer’s protocol. The total RNA or protein were extracted 48 h after transfection and used for the RT-PCR or Western blot analyses.

### 4.9. Scratch Wound Healing Assay

Cell migration potential was evaluated using the wound healing assay. Briefly, DLD-1 or HCT116 cells dissociated from SP-derived DLD-1 or HCT116 tumorspheres (Sp) were seeded onto 6-well plates (Corning Inc., Corning, NY, USA) with complete growth media containing 10% FBS, and cultured to 99–100% monolayer confluency. The cell monolayers were scratched with a sterile yellow pipette tip to denude the culture wells. The cell migration images were captured at the 0 and 24 h time points after denudation, under a microscope with a 10× objective lens, and analyzed with the NIH ImageJ software (https://imagej.nih.gov/ij/download.html).

### 4.10. Transwell Matrigel Invasion Assay

Using the 24-well plate Transwell matrigel invasion system, 3  ×  10^4^ DLD-1 or HCT116 SP cells were seeded into the upper chambers of the inserts (BD Bioscience, 8 μm pore size) containing FBS-free media with different concentrations of 4-AAQB; media containing 10% FBS in the lower chamber served as a chemo-attractant. After 24 h cell incubation, media were discarded; cells on filter membrane were fixed with 3.7% formaldehyde for 1 h, and then stained with 0.2% (*w*/*v*) crystal violet solution, for 15 min, while cells on the upper side of the inserts were gently removed with a cotton swab. The invaded cells were visualized, and the invasive capacity was evaluated as the total number of cells on the lower surface of the membrane, as determined by microscopy.

### 4.11. Colony Formation Assay

2 × 10^4^ DLD-1 or HCT116 cells were plated into 6-well cell culture plates (Corning Inc., Corning, NY, USA) and incubated for 2 weeks at 37 °C after treatment with different concentrations of 4-AAQB. Then, cells were washed thrice with PBS, fixed with ice-cold methanol, stained with 0.005% crystal violet, washed with PBS, and dried at room temperature. Formed colonies were then assessed and counted under microscope. In each well, the total number of colonies with diameter ≥ 100 μm was counted over six randomly selected fields in triplicate assays.

### 4.12. Tumorsphere and Clonal Tumorsphere Formation Assay

5 × 10^3^ DLD-1 or HCT116 cells were plated into wells containing stem cell media with different concentrations of 4-AAQB in ultra-low attachment 6-well plates (Corning Inc., Corning, NY, USA) in quadruplicate. The stem cell media consisted of serum-free growth media, supplemented with 1 × B27 supplement, 10 ng/mL human basic fibroblast growth factor (bFGF; Invitrogen, Grand Island, NY, USA) and 20 ng/mL epidermal growth factor (EGF; Invitrogen, Grand Island, NY, USA), and was changed every 72 h. After 12 days of culture, primary colonospheres consisting of ≥20 cells were counted, and images acquired. Secondary colonospheres were generated by dissociating primary colonospheres by trypsinization,and pipetted through a 22G needle to obtain a single-cell suspension (Thermo Fisher Scientific Inc.). After dissociation of the primary tumorspheres, cell seeding and exposure to 4-AAQB was akin to that for primary tumorspheres.

### 4.13. Immunohistochemistry and Immunofluorescence Staining

Immunohistochemical (IHC) analyses of the Taipei Medical University-Shuang Ho Hospital CRC cohort consisting of different tumor stage colorectal cancer specimens with different clinical stages was performed. Recommendations of the Declaration of Helsinki for biomedical research involving human subjects were followed. Ethical approval for the study was obtained from Joint Institutional Review Board of the Taipei Medical University (approval number: N201602054). A review of patients’ clinical records to determine tumor stage at the time of diagnosis and outcome was carried out. Antibodies against SOD2 (1:200, ab13533 Rabbit pAb) were used in accordance to standard IHC procedure. SOD2 expression was evaluated and scored by two independent pathologists using the quick-score (Q-score) based on the staining intensity (I) and stained cells percentage (P). Staining intensity was defined as 0 (no staining), 1+ (weak), 2+ (moderate), and 3+ (strong). Q=I×P where the maximum score was 300. For the immunofluorescence (IFC) staining, untreated or 4-AAQB-treated DLD-1 or HCT116-derived colonospheres were plated onto poly-*L*-lysine-coated glass cover-slips, fixed with 4% paraformaldehyde, and carefully washed thrice with PBS. This was followed by cell permeabilization with 0.1% Triton X-100/PBS solution for 10 min, and incubation with primary antibodies against Oct-4 and Sox2 (Santa Cruz, CA, USA), then with goat anti–mouse Alexa Fluor488 secondary antibodies (Cat. #: R37120, Thermo Fisher Scientific Inc.). Cell nuclei were labeled with DAPI (4′,6-diamidino-2-phenylindole; Cat. #: D1306, (Molecular Probes, Thermo Fisher Scientific Inc.). Cell visualization and imaging was done using the Nikon E800 fluorescent microscope.

### 4.14. RNA Extraction, Real-Time Polymerase Chain Reaction (RT-PCR) 

Total RNA was isolated from the CRC cell line DLD-1 and resected xenograft tumor sample, using the RNeasy kit (Qiagen Inc., Gaithersburg, MD, USA). The miRNEasy kit (Qiagen) was used for miRNA purification. Total RNA concentration was determined using NanoDrop ND1000 spectrophotometer (Nyxor Biotech, Paris, France). The PCR mixtures were prepared using the SYBR Green Master Mix (Applied Biosystems, Life Technologies, Grand Island, NY, USA). The PCR contained the primers, the fluorogenic probe mix, and the TaqMan Universal PCR Master mix (Applied Biosystems). Amplification reactions were performed in triplicate from 20 ng complementary DNA (cDNA) using the Bio-Rad C1000 real-time PCR system (Bio-Rad, Cambridge, MA, USA) using the following conditions: 95 °C for 3 min, 35 cycles at 95 °C for 15 s, 60 °C for 30 s, 72 °C for 30 s, and 72 °C for 10 min. Results were analyzed, and all values were normalized to the levels of the housekeeping gene β-actin, which served as the internal control. All procedures were in accordance to the manufacturers’ instructions. The PCR primer sequences were as follows: SOD2 (forward): 5′-GCCTCCCTGACCTGCCTTAC-3′, (reverse): 5′-GTGATTGATATGGCCCCCG-3′; hsa-miR-324-5p (forward): 5′-CGCGGATCCGGGTGGATGTAAGGGATGAG-3′; (reverse): 5′-CCGGAATTCTTGGGCTGATCCAGGAGAAG-3′; and β-actin (forward): 5′-CCCTAAGGCCAACCGTGAA-3′, (reverse): 5′-CCAGAGGCATACAGGGACAAC-3′

### 4.15. Oral Administration of 4-AAQB In Vivo and Primary Tumor Experiments 

Severely immunocompromised NOD/SCID mice (6 weeks old) were purchased from BioLASCO (Taipei, Taiwan) and housed in Memorial Hospital Animal Center under SPF conditions. All the animal experiments were performed with strict adherence to the regulations set by MacKay Memorial Hospital Animal Center (Approved protocol number: MMH-104-024). The mice were bred and maintained in pathogen-free conditions, with sterile water and feed, in air-controlled rooms with a specifically designed 12 h light/dark cycle. 1 × 10^5^ DLD-1 cells suspended in 100 μL PBS were injected subcutaneously into the right flank of 6–8-week-old, female NOD/SCID mice (*n* = 5/treatment group; average body weight, 22.5 ± 3.74 g). Tumor growth was monitored daily, and tumor size was measured every 48 h with calipers based on the formula: $\left(x\times y^{2}\right)/2$ , where *x* = longest diameter, and *y* = diameter perpendicular to *x*. When tumors became palpable (~50–150 mm^3^) on day 7–8 after inoculation, the mice were randomly assigned to treatment groups, with oral gavage of vehicle (100 mL 50 mM PBS), 4-AAQB (*n* = 5; 5 mg/kg q48h), FOLFOX (*n* = 5; 15 mg/kg 5-FU q24h, 5 mg/kg folinate q24h and 5 mg/kg oxaliplatin q1wk), or 4-AAQB + FOLFOX (*n* = 5), for five weeks. Tumors were resected and weighed after humane sacrifice of the mice. The feeding habits, motor activity and body weights of the mice were all monitored as indicators of murine general health. Analysis of variance (ANOVA) was used to establish differences between groups, and significance levels were determined by a non-parametric Kruskal-Wallis test. 

### 4.16. Statistical Analysis

All assays were performed at least thrice in triplicates. Values were expressed as mean ± standard error of mean (SEM). Comparison between two groups was estimated using a 2-sided Student’s *t*-test, while a one-way analysis of variance (ANOVA) was used for comparison between three or more groups. All statistical analyses were performed utilizing the GraphPad Prism 5 software (GraphPad Software Inc., La Jolla, CA, USA). *p*-value < 0.05 was considered to be statistically significant.

## 5. Conclusions

In conclusion, this present study demonstrates that 4-AAQB inhibits the aberrant expression of SOD2 through the re-expression of hsa-miR-324-5p, the negative modulation of pluripotency transcription factors, the augmentation of the BAX/BCL-xL ratios, and the reprogramming of malignant colorectal cancer cells from the aggressive mesenchymal phenotype to a relatively benign epithelial phenotype, subsequently enhancing the sensitivity of the cancer cells to conventional chemotherapy and facilitating better prognosis, as depicted in our Schematic abstract.

## Supplementary Materials

The following are available online at https://www.mdpi.com/2072-6694/10/8/269/s1, Figure S1: Bioinformatics-based systemic pharmacoanalyses reveal health effects of 4-AAQB. Predicted values of the health benefits of 4-AAQB are shown in several human systems, with the highest (~99%) health effects in the gastrointestinal system, which includes the colon and rectum, using the ACD/Labs 2.0 v5.0.0.184 platform, Figure S2: 4-AAQB dose determination and ADMET analyses for in vivo assays. (A) Bioinformatic computation of the physicochemical descriptors and prediction of ADME parameters, pharmacokinetic properties, drug-like nature and medicinal chemistry friendliness of 4-AAQB using the SwissADME platform. (B) Data showing the results of in silico drug safety/toxicity analysis on the pkCSM platform. Charts showing (C) the administration route and bioinformatics-based predicted lethality dose, LD in different species, and (D) Predicted values for oral acute toxicity hazard categories, OECD ranges, using the ACD/Labs 2.0 v5.0.0.184. ADMET: absorption, distribution, metabolism, excretion, and toxicity; OECD: Organisation for Economic Cooperation and Development; Cat.: category.
