# Correction: Bamodu et al. 4-Acetyl-Antroquinonol B Suppresses SOD2-Enhanced Cancer Stem Cell-Like Phenotypes and Chemoresistance of Colorectal Cancer Cells by Inducing hsa-miR-324 re-Expression. *Cancers* 2018, *10*, 269

**DOI:** 10.3390/cancers17203304

**Published:** 2025-10-13

**Authors:** Oluwaseun Adebayo Bamodu, Ching-Kuo Yang, Wei-Hong Cheng, David T.W. Tzeng, Kuang-Tai Kuo, Chun-Chih Huang, Li Deng, Michael Hsiao, Wei-Hwa Lee, Chi-Tai Yeh

**Affiliations:** 1 Department of Hematology and Oncology, Cancer Center, Taipei Medical University-Shuang Ho Hospital, New Taipei City 23561, Taiwan; 16625@s.tmu.edu.tw (O.A.B.); 13520@s.tmu.edu.tw (W.-H.C.); 2 Department of Medical Research & Education, Taipei Medical University-Shuang Ho Hospital, New Taipei City 23561, Taiwan; 3 Division of Colorectal Surgery, Department of Surgery, Mackay Memorial Hospital, Taipei City 110, Taiwan; yangchingkao@yahoo.com.tw; 4 School of Life Sciences, The Chinese University of Hong Kong, Hong Kong 153254, China; allqwdd@gmail.com; 5 Division of Thoracic Surgery, Department of Surgery, School of Medicine, College of Medicine, Taipei Medical University, Taipei City 110, Taiwan; doc2738h@gmail.com; 6 Division of Thoracic Surgery, Department of Surgery, Shuang Ho Hospital, Taipei Medical University, New Taipei City 23561, Taiwan; 7 Department of Appiled Chemistry, Chaoyang University of Technology, Taichung 41147, Taiwan; john@newbellus.com.tw; 8 Beijing Bioprocess Key Laboratory, College of Life Science and Technology, Beijing University of Chemical Technology, Beijing 100029, China; dengli@mail.buct.edu.cn; 9 Amoy-BUCT Industrial Bio-Technovation Institute, Amoy 361022, China; 10 Genomics Research Center, Academia Sinica, Taipei 11529, Taiwan; mhsiao@gate.sinica.edu.tw; 11 Department of Pathology, Taipei Medical University-Shuang Ho Hospital, New Taipei City 23561, Taiwan; 12 Department of Pathology, School of Medicine, College of Medicine, Taipei Medical University, Taipei City 110, Taiwan

## Figure

In the original publication [1], there was a mistake in Figure 7A; the control panel of DLD1 cells at 0 h was inadvertently assembled using an incorrect image. This error originated during figure preparation and was not due to the editorial office. The incorrect figure is attached below:


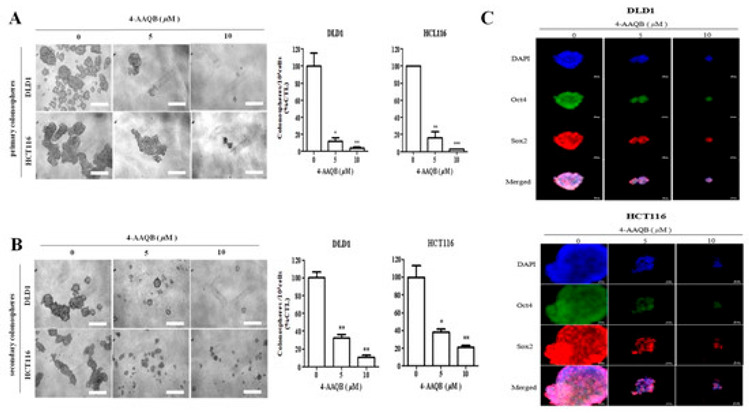


We provide the **corrected Figure 7A** below. This correction does not affect the data interpretation or conclusions of the study.

The authors apologize for any inconvenience caused and state that the scientific conclusions are unaffected. This correction was approved by the Academic Editor. The original publication has also been updated.

## Figures and Tables

**Figure 7:**
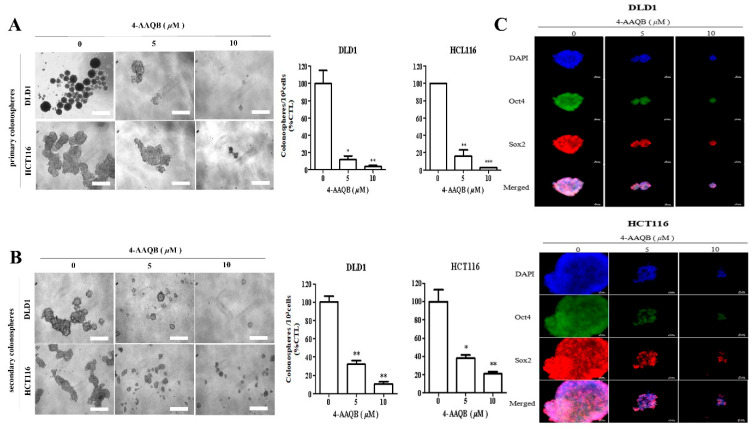
4-AAQB suppresses the cancer stem cell-like phenotype of colorectal cancer cells. (**A**) DLD1 and HCT116 cells pre-treated with 5 or 10 µM 4-AAQB for 48 h exhibit a decreased colonosphere size (**left panel**) and number (**right panel**) in both primary (**A**) and secondary (**B**) generation tumorspheres. (**C**) Treatment with 4-AAQB attenuated OCT4 and SOX2 immunoreactivity and inhibited their nuclear co-localization in DLD1- or HCT116-derived colonospheres using the immunofluorescent (IFC) staining technique. Data are expressed as the mean ± S.E.M. and are representative of experiments performed in triplicate. * *p* < 0.05, ** *p* < 0.01, *** *p* < 0.001 compared with untreated control.
